# Construct Validity of an Obesity Risk Screening Tool in Two Age Groups

**DOI:** 10.3390/ijerph14040419

**Published:** 2017-04-14

**Authors:** Karissa L. Peyer, Greg J. Welk

**Affiliations:** 1 Department of Health and Human Performance, University of Tennessee at Chattanooga, 518 Oak Street, Metro 105 Chattanooga, TN 37403, USA; 2 Department of Kinesiology, Iowa State University, 257 Forker Building, Ames, IA 50011, USA; gwelk@iastate.edu

**Keywords:** Family Nutrition and Physical Activity screening tool, obesity risk factors, socioeconomic status, youth and adolescence

## Abstract

Home environment influences child health, but the impact varies as children move into adolescence. The Family Nutrition and Physical Activity (FNPA) screening tool has been used to evaluate home environments, but studies have not compared the utility of the tool in different age groups. The purpose of this study was to examine the efficacy of the FNPA tool in first and tenth grade samples. Parents of first grade (*n* = 250) and tenth grade (*n* = 99) students completed the FNPA and results were linked to body mass index (BMI) data. FNPA scores were examined by gender, income, race, and school-level socioeconomic status (SES). Correlations examined associations between FNPA scores and several BMI indicators. Logistic and linear regression analyses evaluated the construct validity of the FNPA in both groups. Mean FNPA score differed by age group, by SES in both age groups, and by race in the first grade sample only. Correlations between FNPA score and BMI indicators were higher in the first grade sample, but SES was significantly associated with BMI only in tenth graders. The FNPA has stronger utility in younger children, while school SES is a stronger predictor of adolescent weight status.

## 1. Introduction

The overall prevalence of childhood obesity has been largely stable since 2003–2004, but different trends are evident for children and adolescents. For example, between 2004 and 2012, the prevalence of obesity decreased in young children (from 18.8% to 17.7%), but increased in older adolescents (from 17.4% to 20.5%) [1]. Evidence suggests that there are also disparities in patterns of obesity across ethnic groups with higher prevalence of obesity among Black and Latino children than in White children [2].

These discordant patterns suggest that different factors may play a role in the prevalence of overweight and obesity in these different age groups. Parenting behaviors and the home environment play a critical role in the adoption of lifestyle behaviors in youth [3,4,5,6,7,8,9,10,11,12,13,14], but evidence suggests the effect is weaker as children move into adolescence [15,16,17]. Davison and colleagues proposed a model based on Ecological Systems Theory to explain how child physical activity, dietary intake, and sedentary behavior are influenced by parent behaviors and societal characteristics [18], but screening methods are needed to identify these behavioral and environmental risks before youth become overweight or obese [19,20].

The Family Nutrition and Physical Activity (FNPA) screening tool has been shown to have utility for identifying home environments that may increase a child’s risk for overweight and obesity [21]. The FNPA survey has been used in a number of studies and populations [22,23,24,25,26,27,28], including as a clinical screening tool to facilitate counseling and early intervention [27]. Previous research has supported both the construct [21] and predictive validity [22] of the FNPA in young children; however, it has not been examined in older youth. Because of the documented differences in trends [1] and parenting influence [15,16,17], it is important to directly examine the relationship between the FNPA and weight status among different age groups. Small, but potentially important changes have been made to the FNPA since the original validation work was conducted, so it is also important to re-evaluate the utility of the FNPA in a new cohort of first graders. Thus, the purpose of this study is to evaluate the relative strengths of associations between FNPA and body mass index (BMI) in two separate ages: first grade (~7 years old) and tenth grade (~15 years old) youth. By comparing associations in first graders with a sample of older children from the same district it is possible to directly determine if the FNPA has differential utility by age.

## 2. Materials and Methods

### 2.1. Design and Sample

The study was conducted through an ongoing participatory research agreement with a large Midwestern school district. As part of the process, physical education teachers provide de-identified data on health-related fitness based on the established FITNESSGRAM testing protocol [29]. The battery includes reports of BMI as well as other health related fitness data. The present study examines only height and weight, since focus is on the changes in BMI. Height was measured using a Charder Medical HM200P stadiometer (Taichung City, Taiwan) and weight was measured using a digital scale (Omron SC100, Kyoto, Japan).

Parents of students in first grade and tenth grade were contacted to complete the FNPA and demographic surveys and to allow their child’s BMI data to be merged via student ID. For the first grade sample, parents were recruited via email and hardcopy letter sent to all parents of first grade students. These letters provided a link to the online Informed Consent, FNPA survey, and demographic surveys. For the tenth grade sample, parents were recruited with mailed survey packets sent to their home. The mailer included information about their child’s past involvement in our original study on the FNPA when their child was in first grade [21] as well as information about the importance of capturing data on their current tenth grade student. Demographics in both samples included parent age, gender, race, and education level; household income; and height and weight for both mother and father.

School-level participation (% of students) in the national free- and reduced price lunch program was obtained from publicly available sources and used as a proxy for school-level socioeconomic status (SES).

### 2.2. Description of the FNPA Tool

The FNPA screening tool contains 20 items reflecting ten constructs or topic areas that have been identified as risk factors for overweight/obesity. The constructs include Family Meals, Family Eating Practices, Food Choices, Beverage Choices, Restriction/Reward, Screen Time, Healthy Environment, Family Activity, Child Activity, and Sleep Routine. The updated version of the FNPA assesses the frequency with which each behavior is performed using a four-point Likert scale with options “Never/Almost Never,” “Sometimes,” “Often,” and “Very Often/Always.” For the majority of the screening items, Almost Always/Always is the preferred response and is scored as a 4 while the lowest scoring response (1) is Never/Almost Never. Six items are reverse scored with Never/Almost Never being the preferred response. The total FNPA score is calculated by summing scores. In addition to the total FNPA score, scores for each of the ten constructs are created by summing the scores for the two items within that construct.

### 2.3. Data Processing

Student BMI data from FITNESSGRAM was merged with the individual FNPA scores using student ID numbers. BMI percentile (BMI%), based on age- and gender-specific reference values, was first calculated from gender, height, weight, and age at test date using standard Centers for Disease Control and Prevention (CDC) Statistical Analysis Software SAS codes [30]. There are known limitations in using BMI percentiles for this type of evaluation due to the flattening of the BMI% curve at higher weight status and the differential widths between centiles [31,32]. Therefore, weight status was also computed using an alternative index called Percent Over BMI, which avoids this limitation [32]. This index computes the relative distance of an observed BMI from a given standard and is increasingly used in youth obesity research [33,34,35,36]. In the present study, we computed the relative difference between an individual’s measured BMI and the BMI value for the 50th percentile for age and sex (BMI50).

In both age groups, parent BMIs were also calculated to capture familial risk for overweight and obesity. Underweight and normal-weight parents received a score of zero, overweight parents a score of 1, and obese parents a score of 2. These scores were then summed to create a total a composite risk score for each child (pa).

### 2.4. Data Analyses

Descriptive analyses were used to summarize the weight status and demographic characteristics of boys and girls in the first and tenth grade samples. The prevalence of overweight (≥85th BMI percentile) and obesity (≥95th BMI percentile) were calculated for both age groups. Correlation analyses were used to examine associations between FNPA constructs and the two different BMI indicators. Scores for the ten individual FNPA constructs by gender, family income, school SES, and race categories were also reported but were not statistically evaluated due to concern for excessive comparisons and alpha inflation. Logistic regression was used to evaluate the construct validity of the FNPA in both age groups. For these analyses, children in each grade were grouped into tertiles based on total FNPA score to evaluate whether there were significant differences in odds of overweight/obesity (BMI% ≥ 85%) between children in the highest and lowest tertiles of FNPA score. The primary analyses were conducted using mixed-model regression analyses (SAS PROC GLIMMIX, SAS 9.4, SAS Institute, Inc., Cary, NC, USA) to examine the influence of school SES, parent risk, family income, sex, race/ethnicity, and FNPA score on child weight status. The analyses were conducted with both age groups and with both BMI indicators (BMI% and BMI50) to provide a thorough comparison between the two samples.

### 2.5. Ethics Statement

All subjects gave their informed consent for inclusion before they participated in the study. The study was conducted in accordance with the Declaration of Helsinki, and the protocol was approved by the Institutional Review board at Iowa State University (14-372).

## 3. Results

### 3.1. Comparisons of the Sample Demographics

Descriptive statistics for body mass, height, BMI, BMI percentile, and weight distributions by age group are shown in Table 1. In first graders, survey and anthropometric data were available for 250 students with similar samples for males (*n* = 128) and females (*n* = 122). Of this sample, 81.5% of students were White/Caucasian, 46.2% of parents reported having a Bachelor’s degree or higher, and 21.7% of families had an annual household income of $70,000 or more. Due to small samples of minority racial/ethnic groups, all participants identifying as a group other than White/Caucasian were combined into a non-White group (18.55%) for further analyses. In the tenth grade sample, survey and anthropometric data were available for 99 students: males (*n* = 53) and females (*n* = 46). Of this sample, 78.6% of students were White/Caucasian, 42.3% of parents reported having a Bachelor’s degree or higher, and 20.7% of families had an annual household income of $70,000 or more. As was done with the first grade sample, all participants identified as a racial/ethnic group other than White/Caucasian were combined into a non-White group (21.43%).

### 3.2. Weight Distributions

The distribution of weight categories by age group and gender are provided in Table 1. Collapsing across genders, 12.40% of the first grade students were classified as obese and 30.0% of students were classified as either overweight or obese, slightly below documented trends nationwide (18.6% obese and 31.8% overweight/obese [1]. No differences in the prevalence of overweight or obesity were seen when comparing White to non-White children (*p* = 0.88) or when comparing income groups (*p* = 0.49). Consistent with national data [37], 61.6% of mothers and 73.2% of fathers were either overweight or obese.

Collapsing across genders in the tenth grade sample, 37.73% of students were classified as either overweight or obese, exceeding documented nationwide trends for obesity [1]. There were no significant differences in prevalence of overweight or obesity between White and non-White adolescents (*p* = 0.78) or between income groups (*p* = 0.82). This sample of parents was slightly leaner than national averages [37], with 48.48% of mothers and 67.67% of fathers being either overweight or obese.

### 3.3. Descriptive Analyses of FNPA Scores

Descriptive results for FNPA scores are summarized in Table 2 (a) and (b), including gender, income, and school SES comparisons. In general, FNPA scores were higher for first graders (mean = 65.6) than for tenth graders (mean = 57.5).

There were moderate and significant correlations among the various FNPA constructs showing some clustering of behaviors and environments (Table 3). For example, in first graders, there were significant correlations among the various nutrition constructs and also among the various physical activity constructs, but weaker associations between areas. Patterns of correlations among constructs in the tenth grade sample were similar to those in the first grade sample.

### 3.4. Associations between FNPA and BMI Indicators

One goal in the study was to examine and compare associations between FNPA scores and BMI distributions in both age groups. In first graders, FNPA score was not significantly correlated with BMI% (r = −0.09, *p* = 0.14), but showed a stronger relationship with BMI50 (r = −0.17, *p* = 0.01). Similar patterns were evident in tenth graders, with a low correlation between FNPA and BMI% (r = −0.07, *p* = 0.46), and a slightly stronger association between FNPA and BMI50 (r = −0.19, *p* = 0.06). Correlations were high between the BMI% and BMI50 indicators in both first graders (r = 0.78, *p* < 0.001) and tenth graders (r = 0.82, *p* < 0.001).

To further examine associations between FNPA scores and BMI, the sample was stratified into tertiles based on FNPA scores for logistic regression analyses. In first graders, the prevalence of obesity was significantly higher in children in the lowest tertile of FNPA score (least healthy home environment) (prevalence = 21.25%) compared to children in the highest tertile of FNPA scores (prevalence = 5.97%). Logistic regression showed significantly higher odds for overweight/obesity (odds ratio (OR) = 2.49, confidence interval (CI): 1.17–5.31) in children with FNPA scores in the lowest tertile compared to children in the highest tertile. Because additional factors may influence the home environment and obesity risk, additional covariates were added to the model, including school-level SES, race, and parent risk. The addition of these factors rendered the influence of FNPA insignificant (*p* = 0.36), largely due to the parent risk score which carried some variance in the model (*p* = 0.06). Children with two normal-weight parents (OR = 0.24, CI: 0.08–0.75) or one overweight and one normal-weight parent (OR = 0.33, CI: 0.13–0.84) had significantly lower odds of overweight/obesity compared to children with two obese parents. Contrary to associations in first grade students, FNPA score was not associated with the odds of overweight/obesity in tenth grade students. The addition of school SES, race, and parent risk revealed that students attending the lowest SES schools had higher odds of being overweight/obese compared to students attending the highest SES schools (OR = 5.06, CI: 1.54–16.59).

The primary analyses involved mixed-model regression analyses to more directly evaluate the relationship of school SES, sex, race, parent risk, income, and FNPA score with BMI50 and BMI%. The full results can be found in Table 4. Only parent risk was a significant predictor of BMI50 in first graders (*p* = 0.009). Post-hoc comparisons using a Tukey-Kramer correction showed that there was a significant difference in BMI50 between children with two obese parents (parent risk = 4) compared to those with only one overweight parent (parent risk = 1). Evaluating the same predictors for associations with BMI% resulted in no significant predictors in first grade students. 

The BMI50 model for tenth graders revealed a significant association between school SES and BMI50 (*p* = 0.044) but no other significant predictors. The influence of school SES was less in the BMI% model (*p* = 0.06).

## 4. Discussion

The study examined the utility of the FNPA screening tool in two age groups to identify home environments that increase an individual’s risk for obesity. The study supported findings from the original FNPA [21], since similar associations were observed with the first grade sample tested in the current study. For example, children with an FNPA score in the lowest tertile were found to have increased odds for overweight/obesity compared to children in the highest tertile both in the original study (OR = 1.7, CI: 1.07–2.80) and in the current evaluation (OR = 2.49, CI: 1.17–5.31). The inclusion of parent weight status rendered the FNPA tertile non-significant in both studies; however, it is important to note that the relationship between FNPA and odds of overweight/obesity appears stronger in the current study, suggesting that changes to the FNPA since its original development may have strengthened the ability of the tool to identify risk for overweight.

The correlations between BMI50 and FNPA scores in both age groups (first grade: r = −0.17; tenth grade: r = −0.15) were similar to the relationship found with raw BMI in the original validation of the FNPA (r = −0.17, *p* < 0.01). However, it is worth noting that, in the current study, these relationships were not as strong when using BMI% as the weight status variable. Because the original validation used raw BMI for most analyses, it is possible that the lack of relationship seen with BMI% is due to the different distributions with the various BMI indicators. Raw BMI and BMI50 allow clearer distinctions to be seen between children at the high end of the BMI distributions, whereas children of different raw BMI may become tightly clustered when converting to BMI%.

Rates of child obesity and overall child health are known to be inversely associated with family income as well as aggregate neighborhood socio-economic status [38,39,40]. There is evidence of an increase in disparities in obesity rates between socioeconomic groups over recent years [41,42], and research has shown that high family income does not protect against the risk of obesity conferred by living in a high-deprivation neighborhood [40]. Individuals living in areas that report high levels of distress show higher prevalence of childhood obesity [43]. These findings were supported in the present study, with significant influence of school SES on BMI50 in tenth grade students. Additionally, scores on the Food Choices and Screen Time constructs were most likely to differ between SES and income groups, suggesting that these may be the areas of the home environment most influenced by economic deprivation. While higher obesity levels are frequently documented in Hispanic and non-Hispanic Black children [1,42,44], no differences by racial/ethnic group were seen in the current study. The increased influence of school SES level on weight status in older children than in younger children may suggest that the exposure to a low income/high distress community may have larger influences over a longer period of exposure.

The current study shows that the FNPA has less utility in older children than in younger children. This is not surprising, due to changes that are seen in family dynamics and the influence of parents as children transition into adolescence and begin to prepare for adulthood [17]. Future work should examine the FNPA at additional ages between those surveyed here to determine when efficacy of the tool may begin to wane.

It should be noted that parent BMI risk had a significant association with BMI50 in first graders. This supports a wealth of previous research that has found strong associations between parent and offspring weight status [45,46,47,48]. This finding highlights the need for a family-based approach to child obesity prevention and treatment. While a portion of the relationship between parent and child weight is likely genetic [49], a portion can also be attributed to shared environment and shared behaviors [50,51].

The current study is strengthened by the collection of child weight status through an objective and systematic manner and the consideration of socioeconomic status at both the family and school level. The use of a screening tool capturing a variety of aspects of the home environment rather than physical activity or diet alone helps to examine the relative influence of socioeconomic status on a number of factors. However, the self-reporting of parent weight status and small sample size, particularly for tenth graders, are limitations that future work should address.

## 5. Conclusions

The current study supports the utility of the updated FNPA to identify overweight and obese youth, although this utility is stronger in younger children. Parent weight status appears to have a strong association with child weight in both age groups, although the relative contribution of genetics and shared environment cannot be determined here and should be examined in future work. There is evidence of a significant socioeconomic influence on the quality of the home environment as it pertains to obesogenic behaviors. Future work should also examine the influence of the school/social environment on children and youth, as this relationship appears stronger in older children.

## Figures and Tables

**Table 1:** Descriptive statistics by age group.

	First Grade			Tenth Grade		
First Graders	All Students (*n* = 250)	Males (*n* = 128)	Females (*n* = 122)	All Students (*n* = 99)	Males (*n* = 53)	Females (*n* = 46)
Body Mass (kg)	25.79 (6.8)	25.70 (7.3)	25.88 (6.3)	71.65 (16.0)	75.47 (15.4)	67.25 (15.6)
Height (cm)	123.22 (5.8)	123.81 (6.0)	122.31 (5.5)	169.53 (10.6)	176.63 (7.2)	161.51 (7.5)
BMI	16.80 (3.4)	16.58 (3.7)	17.03 (3.0)	24.83 (5.1)	24.11 (4.8)	25.66 (5.4)
BMI Percentile	59.30 (30.6)	56.56 (33.7)	62.16 (26.9)	72.80 (26.3)	70.01 (26.6)	76.00 (25.8)
Weight Categories (%)						
Underweight		10.2	1.0		2.0	0.0
Normal Weight		58.9	70.5		60.4	45.7
Overweight		18.0	17.2		20.8	30.4
Obese		13.3	11.5		17.0	23.9

BMI, body mass index. All values are mean (standard deviation) unless otherwise stated.

**Table 2:** (**a**) Family Nutrition and Physical Activity (FNPA) total and construct scores by gender, family income, and school socioeconomic status (SES) level in first grade students; (**b**) FNPA total and construct scores by gender, family income, and school SES level in tenth grade students.

(**a**)												
		**Total FNPA**	**Meals**	**Eating**	**Food Choices**	**Beverages**	**Restriction**	**Screen**	**Environment**	**Family PA**	**Child PA**	**Sleep**
All (*n* = 250)	Mean	65.58	7.75	6.24	6.29	5.87	6.81	5.89	6.90	6.71	5.66	7.38
	*SD*	6.3	0.7	1.0	1.2	1.4	1.0	1.7	0.9	1.2	1.5	1.1
Gender												
Boys (*n* = 128)	Mean	65.41	7.80	6.29	6.36	5.75	6.78	5.98	6.93	6.70	5.53	7.41
	*SD*	6.5	0.6	1.0	1.4	1.6	1.0	1.7	0.9	1.3	1.4	1.1
Girls (*n* = 122)	Mean	65.95	7.70	6.18	6.43	6.00	6.84	5.80	6.87	6.83	5.80	7.33
	*SD*	6.1	0.8	1.1	1.1	1.3	1.0	1.7	1.0	1.1	1.5	1.1
Family Income												
<$20,000 (*n* = 10)	Mean	61.5	7.57	5.62	5.85	5.36	6.33	4.57	6.54	6.64	4.93	7.43
	*SD*	4.5	0.8	1.9	1.5	1.6	1.6	2.0	0.7	1.2	0.9	1.4
$20,000–$40,000 (*n* = 42)	Mean	65.1	7.67	6.40	6.34	5.59	6.96	5.93	6.60	6.66	5.51	7.67
	*SD*	6.7	0.8	1.1	1.4	1.7	1.1	1.7	1.0	1.2	1.6	0.6
$40,000–$70,000 (*n* = 65)	Mean	64.83	7.77	6.00	6.49	5.76	6.86	5.79	6.78	6.74	5.35	7.22
	*SD*	6.8	0.8	1.0	1.1	1.3	1.0	1.8	1.0	1.2	1.6	1.2
$70,000–$100,000 (*n* = 52)	Mean	67.13	7.81	6.43	6.31	6.06	6.87	6.07	7.06	7.06	6.17	7.46
	*SD*	6.4	0.5	0.9	1.2	1.4	0.8	1.6	1.0	1.2	1.4	1.1
>$100,000 (*n* = 61)	Mean	66.33	7.83	6.35	6.52	6.14	6.71	6.08	7.18	6.49	5.85	7.25
	*SD*	5.3	0.5	0.9	1.2	1.4	0.9	1.5	0.7	1.3	1.2	1.1
School SES Tertile												
High School SES (*n* = 85)	Mean	66.45	7.85	6.45	6.52	6.15	6.77	6.23	7.05	6.57	5.79	7.25
	*SD*	5.9	0.5	1.0	1.3	1.3	0.9	1.5	0.8	1.4	1.4	1.1
Medium School SES (*n* = 80)	Mean	66.9	7.82	6.47	6.63	5.89	7.00	6.13	6.98	6.86	5.84	7.41
	*SD*	6.7	0.7	1.0	1.1	1.5	0.9	1.6	1.0	1.2	1.6	1.2
Low School SES (*n* = 85)	Mean	63.57	7.60	5.82	6.05	5.57	6.66	5.33	6.68	6.82	5.37	7.48
	*SD*	5.9	0.9	1.1	1.3	1.4	1.2	1.8	1.0	1.1	1.4	1.0
(**b**)												
		**Total FNPA**	**Meals**	**Eating**	**Food Choices**	**Beverages**	**Restriction**	**Screen**	**Environment**	**Family PA**	**Child PA**	**Sleep**
All (*n* = 99)	Mean	57.5	6.72	5.65	6.06	5.54	6.2	4.05	5.77	5.8	4.5	6.22
	*SD*	7.5	1.4	1.2	1.1	1.2	1.0	1.4	1.4	1.3	1.7	1.5
Gender												
Boys (*n* = 53)	Mean	58.94	7	5.64	6.11	5.55	6.32	4.15	6.0	6.04	5.75	6.38
	*SD*	7.8	1.3	1.2	1.2	1.1	1.0	1.5	1.4	1.3	1.6	1.5
Girls (*n* = 46)	Mean	55.83	6.39	5.65	6.0	5.52	6.07	3.93	5.5	5.52	5.2	6.04
	*SD*	6.9	1.5	1.3	1.1	1.3	1.0	1.4	1.3	1.2	1.8	1.6
Family Income												
<$20,000 (*n* = 8)	Mean	57.5	6.5	5.75	5.63	5.63	6.13	3.88	6.38	6.13	5.0	6.5
	*SD*	9.3	1.8	1.8	1.2	0.7	1.1	1.7	1.2	1.4	1.6	1.7
$20,000–$40,000 (*n* = 16)	Mean	54.63	6.13	5.63	5.75	5.25	5.88	4.00	5.88	5.38	5.06	5.69
	*SD*	8.4	1.6	1.2	1.4	1.2	1.0	1.1	1.5	1.6	2.1	1.8
$40,000–$70,000 (*n* = 22)	Mean	57.5	5.82	5.41	6.0	5.32	6.5	3.95	6.14	5.82	5.14	6.41
	*SD*	6.0	1.3	1.2	0.9	1.2	0.9	1.4	1.2	1.5	1.7	1.2
$70,000–$100,000 (*n* = 19)	Mean	58.21	6.47	5.42	5.74	5.58	6.11	4.32	5.89	6.16	5.79	6.74
	*SD*	6.9	1.6	1.0	0.9	1.1	1.0	2	1.1	1.1	1.7	1.3
>$100,000 (*n* = 27)	Mean	59.3	7.15	6.08	6.56	6.04	6.37	4.11	5.44	5.74	6	5.81
	*SD*	8.3	1.2	1.2	1.2	1.2	1.0	1.3	1.7	1.2	1.5	1.7
School SES Tertile												
High School SES (*n* = 30)	Mean	59.73	7.2	6	6.53	5.9	6.37	4.43	5.73	5.77	5.77	6.03
	*SD*	6.6	1.2	1.1	1.1	1.0	1.0	1.3	1.6	1.2	1.6	1.7
Medium School SES (*n* = 36)	Mean	58.17	6.86	5.67	5.86	5.47	6.31	4.17	5.83	5.92	5.61	6.47
	*SD*	7.8	1.4	1.3	1.1	1.1	1.0	1.4	1.3	1.3	1.6	1.4
Low School SES (*n* = 33)	Mean	54.72	6.12	5.3	5.85	5.27	5.94	3.58	5.83	5.7	5.12	6.12
	*SD*	7.5	1.6	1.1	1.1	1.4	0.9	1.5	1.4	1.5	1.9	1.6

PA: physical activity; SD: standard deviation.

**Table 3:** Correlations among FNPA constructs in both age groups.

		Correlations among FNPA Constructs for First Graders										
		FNPA Score	Meals	Eating	Food Choices	Beverage	Reward and Restrict	Screen Time	PA Environ	Family PA	Child PA	Sleep
Correlations Among FNPA Constructs for Tenth Graders	FNPA score	1	0.39 *	0.50 *	0.65 *	0.51 *	0.41 *	0.70 *	0.55 *	0.54 *	0.52 *	0.38 *
	Meals	0.53 *	1	0.21 *	0.22 *	0.17 *	0.1	0.15 *	0.18 *	0.17 *	0.12	0.25 *
	Eating	0.39 *	0.09	1	0.43 *	0.22 *	0.20 *	0.43 *	0.19 *	0.03	0.02	0.2
	Food Choices	0.43 *	0.31 *	0.33 *	1	0.29 *	0.23 *	0.39 *	0.26 *	0.28 *	0.17 *	0.17 *
	Beverage	0.45 *	0.15	0.34 *	0.32 *	1	0.06	0.32 *	0.17	0.11	0.05	0.18 *
	Reward and Restrict	0.57 *	0.08	0.34 *	0.25 *	0.32 *	1	0.34 *	0.12	0.08	0.08	0.27 *
	Screen Time	0.66 *	0.27 *	0.24 *	0.18	0.25 *	0.41 *	1	0.30 *	0.23 *	0.22 *	0.17 *
	PA Environ	0.58 *	0.21 *	0.08	0.02	0.07	0.33 *	0.28 *	1	0.46 *	0.31 *	0.08
	Family PA	0.61 *	0.19	−0.03	0.1	0.07	0.29 *	0.28 *	0.51 *	1	0.43 *	0.02
	Child PA	0.65 *	0.29 *	−0.04	0.18	0.14	0.19	0.33 *	0.33 *	0.61 *	1	0.07
	Sleep	0.56 *	0.43 *	0.08	−0.03	0.05	0.24 *	0.38 *	0.39	0.31 *	0.29 *	1

* Indicates significance at *p* < 0.05.

**Table 4:** Results of Mixed Model Regressions.

	First Grade		Tenth Grade	
	BMI%	BMI50	BMI%	BMI50
School SES	0.90	0.80	0.06	0.04 *
Sex	0.35	0.80	0.31	0.11
Race/Ethnicity	0.66	0.55	0.31	0.77
Parent Risk	0.08	0.01 *	0.20	0.15
Income Group	0.49	0.47	0.29	0.56
FNPA	0.55	0.10	0.67	0.36

* Indicates significance at *p* < 0.05; BMI50 = ((child BMI – BMI for 50th percentile)/BMI for 50th percentile) × 100.
